# Understanding the Role of the Gut Microbiome in Diabetes and Therapeutics Targeting Leaky Gut: A Systematic Review

**DOI:** 10.7759/cureus.41559

**Published:** 2023-07-08

**Authors:** Aishwarya Sadagopan, Anas Mahmoud, Maha Begg, Mawada Tarhuni, Monique Fotso, Natalie A Gonzalez, Raghavendra R Sanivarapu, Usama Osman, Abishek Latha Kumar, Lubna Mohammed

**Affiliations:** 1 Internal Medicine, California Institute of Behavioral Neurosciences & Psychology, Fairfield, USA; 2 Internal medicine, California Institute of Behavioral Neurosciences & Psychology, Fairfield, USA

**Keywords:** insulin resistance, type 2 diabetes, glucose metabolism, microbial transplantation, prebiotics, probiotics, gut microbiome, diabetes

## Abstract

The gut microbiota has been studied and continues to be a developing area in the pathognomic development of metabolic diseases like diabetes. Treatment with diet changes, the addition of supplements like prebiotics/probiotics, and the impact of fecal microbial transplantation can be correlated to targeting changes in dysbiosis. Understanding the impacts of various anti-hyperglycemic agents such as metformin and the implications of post-bariatric surgery on the gut microbiota diversity has emerged. These areas of study are crucial to understanding the pathognomic aspects of diabetes disease progression at the microbial level of metabolic and inflammatory mechanisms, which may give more insight into focusing on the role of diet prebiotic/probiotic supplements as potential forms of prospective management in diabetes and the development of more agents that target gut microbiota, which harbors low-grade inflammation. Intestinal dysbiosis was consistently observed in the mechanism of gut microbial change in diabetic individuals, contributing to reduced insulin sensitivity and poor glycemic control. This systematic review was carried out using the Preferred Reporting Items for Systematic Reviews and Meta-analyses (PRISMA) 2020 checklist. We performed a literature search using the PubMed, Google Scholar and Science Direct databases in accordance with the eligibility criteria and ultimately selected 14 articles for final analysis. The Scale for the Assessment of Narrative Review Articles (SANRA) and the PRISMA 2020 checklist were used to assess the quality of selected articles for cross-sectional studies, traditional literature reviews, and systematic reviews, respectively. We collected papers from 2012 to 2022 for this review. We gathered articles from databases, such as this study, which show there is a strong connection between microbiota and diabetes that appears to exist. The objective is to assess and identify any dietary and therapeutic agents that may alter the microbiota and potentially target and modulate insulin sensitivity. This review article will discuss the pathophysiological effects of gut microbiota in diabetes management and the impact of various gut biodiversity therapeutics that can aid in reversing insulin sensitivity.

## Introduction and background

Globally, diabetes is increasing in prevalence and has grown to become the fifth leading cause of death reported in 2019, estimated to reach 548 million by 2045. Of the different forms of diabetes, type 2 diabetes mellitus alone accounts for most cases [1]. Diabetes mellitus is a chronic metabolic disorder characterized by hyperglycemia either due to insulin resistance or insulin deficiency. There are two major types of diabetes, including type 1 diabetes, which is autoimmune-mediated, and type 2 diabetes, which occurs due to multi-genetic and environmental factors [2]. The underlying pathogenesis of diabetes is due to the development of beta cell dysfunction and beta cell destruction in the pancreas, leading to hyperglycemia. Chronically elevated blood glucose levels have been shown to lead to the development of microvascular (retinopathy, neuropathy, and nephropathy) and macrovascular complications (ischemic heart disease, peripheral artery disease, and stroke) with multi-organ involvement, significantly increased morbidity, and mortality [3]. Recent and emerging therapeutic options for diabetes management with anti-diabetic agents and ongoing drug development research have been promising. However, understanding the dynamics of lifestyle nutritional factors and their microbial changes in the gut, observed after consuming a low-glycemic, high-fiber diet, showed evidence of effective glycemic control by insulin signaling and, in some instances, remission of high blood glucose levels [4]. 

Two thousand years ago, Hippocrates said, “All diseases begin in the gut,” a statement accurately associated with immune system dysregulation and susceptibility to disease [5-7]. The gastrointestinal tract has a complex and distinct population of microorganisms that comprise the gut microbiota. We know that diet can change the gut microbiota and may influence metabolism [5]. When the gut microbiota changes in terms of bacterial composition, known as dysbiosis, it predisposes to inflammation, which research demonstrates is the onset of altered gut homeostasis in patients with diabetes [5-7]. Understanding the existing balance of the gut microbiome vs. alterations in the gut microbiome before and after intake of supplements, diet modification, anti-diabetic medications, and fecal microbial transplantation was significant, as the composition of the intestinal microbiota has shown to affect host metabolism and cause abnormal blood glucose regulation [8-10]. As many treatment options target the microbiota, a bidirectional relationship between diabetes and the gut microbiota can be observed. The synthesis of amino acids, production of short-chain fatty acids (SCFA), absorption of nutrients, prevention of colonization with pathological bacteria, influence on the composition of bile acid, and production of several pattern recognition molecules are all ways that the gut microbiota is involved in both healthy and disease states [7,11,12].

Mounting details demonstrate that changes in the human genome, dietary habits, or a reduction in daily physical activity are not alone responsible for the rise in obesity and type 2 diabetes [13-15]. This systematic review looks into possible pathogenesis and approaches to therapeutic options for managing diabetes as it pivots on the onset of low-grade inflammation concerning the gut microbiota. The central perspective of this review is to evaluate the understanding of the associations between gut microbiota and diabetes and to uncover any new therapeutic agents that may modulate the microbiota, as there is a clear relationship between gut microbiota and diabetes.

## Review

Methods and results

The purpose of this systematic review, using Preferred Reporting Items carried out for Systematic Reviews and Meta-analyses (PRISMA) 2020, was to determine the efficacy and advantages of therapeutic targets for type 2 diabetes at the intestinal microbial level. We collected and reviewed articles, including clinical trials, literature reviews, systematic reviews, and meta-analyses published between 2012 and 2022. The databases that were used to collect these articles included PubMed, PubMed Central, ScienceDirect, and Google Scholar. After applying appropriate filters, a total of 421 reports were identified from these four databases. They were further screened and subjected to quality assessment tools, which finally yielded 14 studies that were included in this systematic review. Table 1 shows the search strategy used and the databases used to collect articles. 

**Table 1:** Search strategy and databases that were used to collect articles

Search Terms	Databases Used	Number of Research Papers Identified
Mellitus/diagnosis"[Mesh] OR "Diabetes Mellitus/etiology"[Mesh] OR "Diabetes Mellitus/immunology"[Mesh] OR "Diabetes Mellitus/metabolism"[Mesh] OR "Diabetes Mellitus/microbiology"[Mesh] OR "Diabetes Mellitus/pathology"[Mesh] OR "Diabetes Mellitus/physiopathology"[Mesh] )("Gastrointestinal Microbiome/drug effects"[Mesh] OR "Gastrointestinal Microbiome/genetics"[Mesh] OR "Gastrointestinal Microbiome/immunology"[Mesh] OR "Gastrointestinal Microbiome/physiology"[Mesh] ) ("Gastrointestinal Microbiome/drug effects"[Mesh] OR "Gastrointestinal Microbiome/genetics"[Mesh] OR "Gastrointestinal Microbiome/immunology"[Mesh] OR "Gastrointestinal Microbiome/physiology"[Mesh] ) ("Glucose Metabolism Disorders/etiology"[Mesh] OR "Glucose Metabolism Disorders/metabolism"[Mesh] OR "Glucose Metabolism Disorders/pathology"[Mesh] OR "Glucose Metabolism Disorders/physiopathology"[Mesh] ) ("Gastrointestinal Microbiome/drug effects"[Mesh] OR "Gastrointestinal Microbiome/genetics"[Mesh] OR "Gastrointestinal Microbiome/immunology"[Mesh] OR "Gastrointestinal Microbiome/physiology"[Mesh] )("Glucose Metabolism Disorders/etiology"[Mesh] OR "Glucose Metabolism Disorders/metabolism"[Mesh] OR "Glucose Metabolism Disorders/pathology"[Mesh] OR "Glucose Metabolism Disorders/physiopathology"[Mesh] )	PubMed Central, PubMed	316, 52
Gut Microbiota and Diabetes	Google Scholar	11
Gut Microbiota and Diabetes	Science Direct	31

Quality Assessment Tools

The quality assessment was performed independently by two reviewers. The tools we used are the Scale for the Assessment of Narrative Review Article (SANRA) and the PRISMA 2020 checklist, traditional literature reviews, and systematic reviews, respectively. Each of these appraisal tools has specific criteria to evaluate the studies using a point system. Accordingly, only those articles that have a high quality score of >7 were selected for data extraction.

Data Extraction

The data were extracted using standardized recording tools by two independent reviewers. After we assessed the final number of articles as per the eligibility criteria mentioned above, the content of the selected data was searched for relevant information related to our research topic. Moreover, the information was divided into different subheadings to address the research question in the discussion section.

Eligibility Criteria 

**Figure 1:**
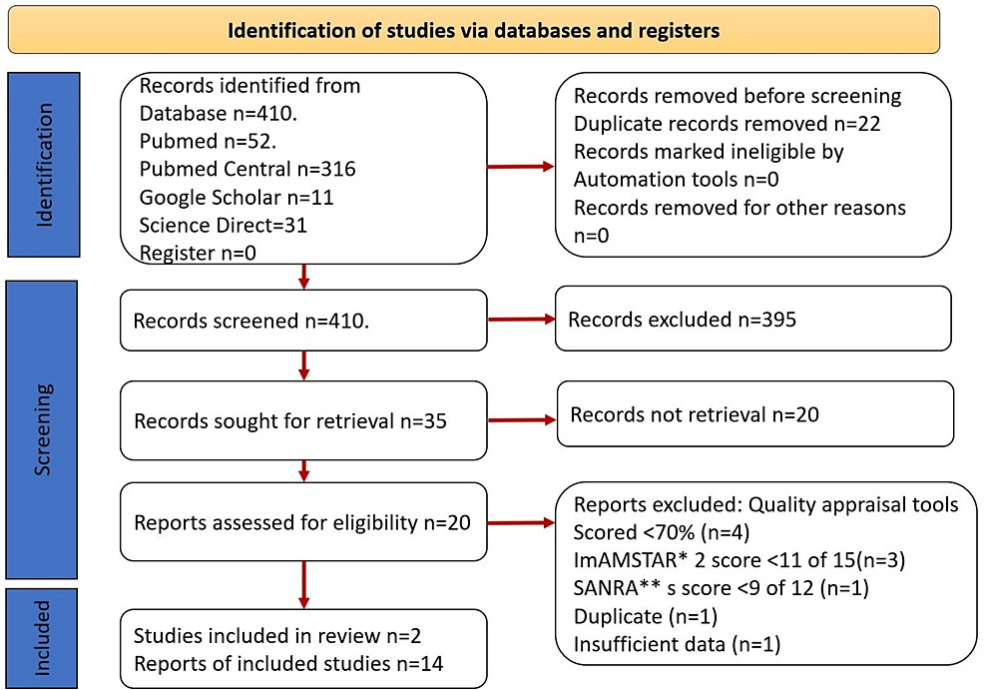
PRISMA flow chart of this systematic review. PRISMA: Preferred reporting items for systematic reviews and meta-analysis

The selection was performed by the authors, who worked independently based on the PICO criteria (population, intervention, comparison, outcome) represented in Figure 1.

Inclusion and Exclusion Criteria

Articles were selected based on the following inclusion criteria related to participants, interventions, and outcomes. (i) papers written and published in English Language, (ii) intervention studies in papers focusing on all age groups, (iii) papers focusing on all ethnicities, (iv) papers focusing on type 2 and type 1 diabetes, (v) papers that are 10 years old or less, (vi) papers that include adults 19 years old plus, (vii) papers with animal studies, and (viii) papers including gestational diabetes individuals. Exclusion criteria were interventions with (i) papers excluding infants and newborns, (ii) ICU patients, (iii) grey literature, (iv) translated papers, (v) publications in other languages than English, and (vi) studies with a publication date greater than 10 years were excluded.

Using different databases with the mentioned search strategies, a total of 410 records were extracted from databases, and different inclusion criteria filters were used to exclude 395 studies. In the next step, 22 duplicate studies were recognized and removed. A total of 410 articles were screened based on title and abstract, and 395 studies were selected to investigate more. Thirty-five studies were subjected to quality assessment by using quality assessment tools such as AMSTAR 2 (for systematic reviews and meta-analyses), and SANRA (for narrative traditional review articles), and a final number of 14 were selected to include in the study. The last search date for the study materials was May 25, 2023. The PRISMA chart, shown in Figure 1, gives an overview of the screening process.

A normal, stable gut microbiome is essential for optimal good health, with the ideal balance of thousands of gram-positive and gram-harmful bacteria phyla and species needed to exist in harmony, such as Bacteroidetes, Firmicutes, and Fusobacteria, to name a few [12,14,15]. When this makeup and functionality change, known as dysbiosis, the function of the microbiome is revised, which leads to changes in the intestinal microvilli, immune system mission, defense against pathogens, and the breakdown of non-digestible polysaccharides and metabolism itself, which leads to a state of chronic inflammation [11,16,17]. With an ongoing form of inflammation, obesity, pre-diabetes, and type 2 diabetes have been shown to have a similar association with dysbiosis in the gut. This is depicted by a reduction in gut biodiversity, higher energy consumption ability in the large intestine of obese individuals, and the maintenance of a systemic inflammatory state, which is leading to increased insulin resistance [18,19].

Pathophysiology of normal gut microbiome and dysbiosis

The stable gut microbiome starts growing its diversity under the influence of initial exposure from the vaginal microbiome after delivery, exclusive breastfeeding or formula feeding, and exposure to antibiotics in the neonatal period [16,19]. With the intake of a varied diet and environmental exposures, it slowly stabilizes in adulthood, although there is more evidence that the gut microbiome is constantly changing [15,16,18]. The gut bacteria carry out complex dietary food digestion, converting indigestible carbs into energy, vitamin production, and immune cell growth. With aging, the microbiota has been shown to slowly deplete progressively, which raises the question of whether our microbiome is also constantly changing. 

The gut microbiota is important in the digestion of absorbed nutrients, creating barriers against pathogens and toxins, which play a crucial role in immune function. Under conventional physiologically healthy settings, the diversity and behavior of the gut microbiota remain consistent, with certain species like *Bacteroidetes, Firmicutes, Ruminococcus, Lactobacillus, Clostridium, Fusobacteria, Actinobacteria, Verrucomicrobia, and Fusobacteria* functioning as needed as dominant and rare species coexist in harmony [11,14]. The ratio of Firmicutes/Bacteroidetes levels was found to be lower in type 2 diabetes patients in several clinical trials [20,21].

The diet composition containing predominantly carbohydrates and amino acids is fermented into short-chain fatty acids (SCFAs), an important factor in gut integrity that influences signaling pathways in intestinal gluconeogenesis, gut wall integrity, GLP-1 (Glucagon-like peptide 1) secretion, beta cell function, and insulin secretion [22,23]. Similarly, a bile acid pool and collaboration of FXR (Farnesoid X receptor) and TGR5 (G-protein-coupled bile acid receptor) signaling together with FGF19 (Fibroblast growth factor 1) signaling are noteworthy molecular actors in the gut microbiota [12,14,17]. Further, the gut is tightly regulated by tight junction proteins (claudin, ZO-1, and occludin), a healthy endocannabinoid system tone, and the intestinal alkaline phosphatase's Lipopolysaccharides (LPS) system, which all contribute to the maintenance of proper lipid metabolism, inflammatory responses, energy balance, and maintaining the integrity of the gut barrier with the help of PAMPs (pathogen-associated molecular proteins), of which there are four subtypes [11,17,24]. GLUT 2 is vital, as it maintains the gut barrier through zonula occludens-1 (ZO-1) and E-cadherin, which are involved in tight junctions in the intestinal epithelial cells [12,18]. Bile acids, SCFA, and the endocannabinoidome system work collaboratively to help create a healthy gut barrier, which helps reduce food intake, lipids, blood glucose, insulin resistance, inflammation, and hepatic steatosis [17,19,25]. By targeting all these components collaboratively that form the underlying origin of metabolic syndrome, the gut microbiome may have a synergistic role in cardiometabolic risk factor elements [12,14]. 

There are some main species of bacteria, including *Prevotella, Ruminococcus, Bacteroidetes,* and *Firmicutes*. A microbial community imbalance known as dysbiosis is seen in chronic inflammatory diseases such as autoimmune and metabolic disorders. Growing data indicate that intestinal barrier integrity, particularly the mucus layer and epithelial cell junctions, can be impaired by microbial dysbiosis, leading to increased intestinal permeability [26]. These barriers are important as their dysfunction may lead to the leakage of bacteria or products of bacteria, which can start a cascade of chronic inflammation, particularly one that is ongoing, and a state of low-grade inflammation, one that is noted in diabetes [12,13].

Dysbiosis of the gut microbiota is known to affect the production of SCFA, altering the bile acid profile and the endocannabinoid system, causing a reduction in GLP-1, GLP-2 (glucagon-like-peptide 1,2) and PYY (peptide YY), which lead to an impaired gut barrier and pave the way for a cascade of events that lead to decreased insulin sensitivity, increased ongoing inflammation, more oxidative stress, increased steatosis, and increased fat mass [17]. This influences the epigenetic control over genes that govern inflammation and insulin resistance in T2DM. In addition, several phyla of Firmicutes were consistently observed to be lower in the illness groups [14,27].

**Figure 2:**
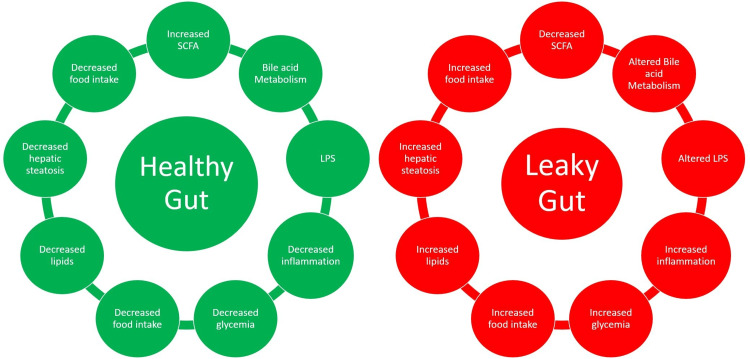
A descriptive figure of a healthy gut model (green model) surrounded by various metabolic mechanisms vs. pathological mechanisms (red model) associated with gut integrity in dysbiosis (leaky gut) noted in diabetic individuals alters molecular mechanisms and interactions with host factors. SCFA: Short chain fatty acids, LPS: Lipopolysaccharides This figure is the author’s own creation.

AMP-activated protein kinase (AMPK), which is known to be involved in the metabolism of cholesterol, lipids, and glucose, is impacted by SCFA in the liver and muscle tissues. GLP-1 has been associated with SCFA via the G-protein-coupled receptor 43 (GPR43) pathway [23]. An increase in SCFAs, which increase GLP-1 and PYY levels, has proven to improve insulin sensitivity in obese and type 2 diabetic individuals [20,21]. The flowchart in Figure 2 is a conceptual model developed to demonstrate the potential aspects associated with a healthy vs. leaky gut (dysbiosis) that are observed in diabetic individuals [20,27,28].

Due to oxidative stress and low-grade inflammation observed in microbial dysbiosis, the pancreatic beta cell death rate was found to increase, and a state of insulin resistance also increased [16,17]. Type 2 diabetes studies revealed by earlier investigations suggest a decrease in Firmicutes. Also noted was a decrease in butyrate-producing bacterial species, such as *Clostridium, Eubacterium rectale, Faecalibacterium prausnitzii, Roseburia intestinalis, *and *Roseburia inulinivorans. Ruminococcus, Fusobacterium, and Blautia genera* have positive correlations with type 2 diabetes, but *Bifidobacterium, Bacteroides, Faecalibacterium, Akkermansia, *and* Roseburia* genera have negative correlations [17,18,28].

Enhanced intestinal LPS permeability in the gut seems to play a central role in chronic inflammation, contributing to reduced insulin sensitivity. In particular, the mucus layer of the gastrointestinal tract appears to be a suitable medium for the growth of bacteria. When this mucus layer is disturbed, which is commonly observed in individuals with a high-fat diet, which is prevalent in diabetic individuals, it may lead to the development of diabetes and other metabolic disorders [24]. 

With the aid of anaerobic bacteria, short-chain fatty acids (SCFAs) like acetate, propionate, butyrate, and lactate, which are produced from undigested food, have an essential effect on metabolic disorders and are favorable for glucose metabolism. It has been linked to stimulating the release of GLP-1 and peptide YY, which regulate glucose metabolism and insulin secretion, by activating gut hormone receptors G protein-coupled receptor (GPR)-41 (aka FFAR3) and GPR-43 (aka FFAR2) [12,26,29]. SCFA concentrations are altered in type 2 diabetes. According to specific investigations, gut dysbiosis in people with type 2 diabetes affects the SCFA concentration significantly [24]. In obesity, which is commonly associated with diabetes, SCFAs were found to have several positive effects on gut metabolism, with increased SCFA production being observed to be responsible for higher energy extraction due to increased diet consumption [26].

In a number of systematic review studies [12,15,26], pre-diabetic and type 2 diabetes patients had less gut variety and diversity than people with normal glucose tolerance. Particularly in newly diagnosed type 2 diabetes, higher levels of the phylum *Firmicutes* and lower levels of the phylum *Bacteroidetes* were found. Similarly, an association with fasting plasma glucose was discovered to be related to *Lactobacillus*. The amounts of microorganisms were further altered by dietary changes [15]. 

The role of diet and prebiotics/probiotic supplementation modulating the gut microbiota

Diet

The composition of the gut microbiota continues to shift based on diet, disease state, genetics, and medication intake [23]. Several studies have shown that many diets, including plant-based, high-fat, and low-fat, change the microbiota composition significantly [23]. Dietary fibers, which are associated with an ideal weight, low insulin resistance, low cholesterol, and optimal blood sugar levels, mainly consist of cellulose, resistant starch, dextrin, inulin, lignin, pectin, glucan, and oligosaccharides [19]. Increases in certain bacteria species decreases in certain species of bacteria, and changes in the ratio of the bacterial composition were all noted with the incorporation of different diets [23]. A crucial part of the dietary fiber makeup in gut microbiota is that they produce metabolites, of which short-chain fatty acids (SCFAs), primarily acetate, propionate, and butyrate, are more noteworthy [19].

A combination of lifestyle factors with changes in diet and exercise has been shown to alter the role of gut microbiota in impacting blood glucose levels in prediabetes and type 2 diabetes [16]. It is unknown how bacterial composition leads to metabolic impairment through diet. Still, it is proposed to be due to altered glucagon-like peptide-1 and -2 levels, increased lipopolysaccharides, ongoing inflammation due to increased oxidative stress, reduced SCFAs, and increased energy usage. By understanding the gut microbiome's dietary patterns, many prospects targeting dietary approaches can be appreciated and researched further [16].

Plant-based diets and animal-based diets clearly showed inverse patterns of physiological inflammatory changes. Plant-based diets demonstrated a decrease in the composition of opportunistic bacteria, which resulted in a decrease in the activation of lipopolysaccharides and inflammatory cytokines. SCFA production has increased in plant-based diets, which has been found to lower inflammation in obesity and diabetes [12,14]. Animal-based and high-fat diets have demonstrated increased bacterial metabolites and activation of opportunistic bacteria, further activating ongoing lipopolysaccharide and inflammatory cytokine activation with reduced SCFA levels over time [20]. 

The Mediterranean diet, which is high in plant-based foods, low in animal protein and saturated fat, and high in fiber and omega-3 fatty acids, was linked to higher concentrations of SCFA, *Prevotella*, and *Firmicutes*, known to break down fiber. Researchers discovered that the ratio of *Prevotella* to *Bacteroides* was higher in individuals who followed the Mediterranean diet consistently over a period of time, showing that a diet rich in natural fiber and resistant starch positively affects the bacterial composition of human beings [20].

**Figure 3:**
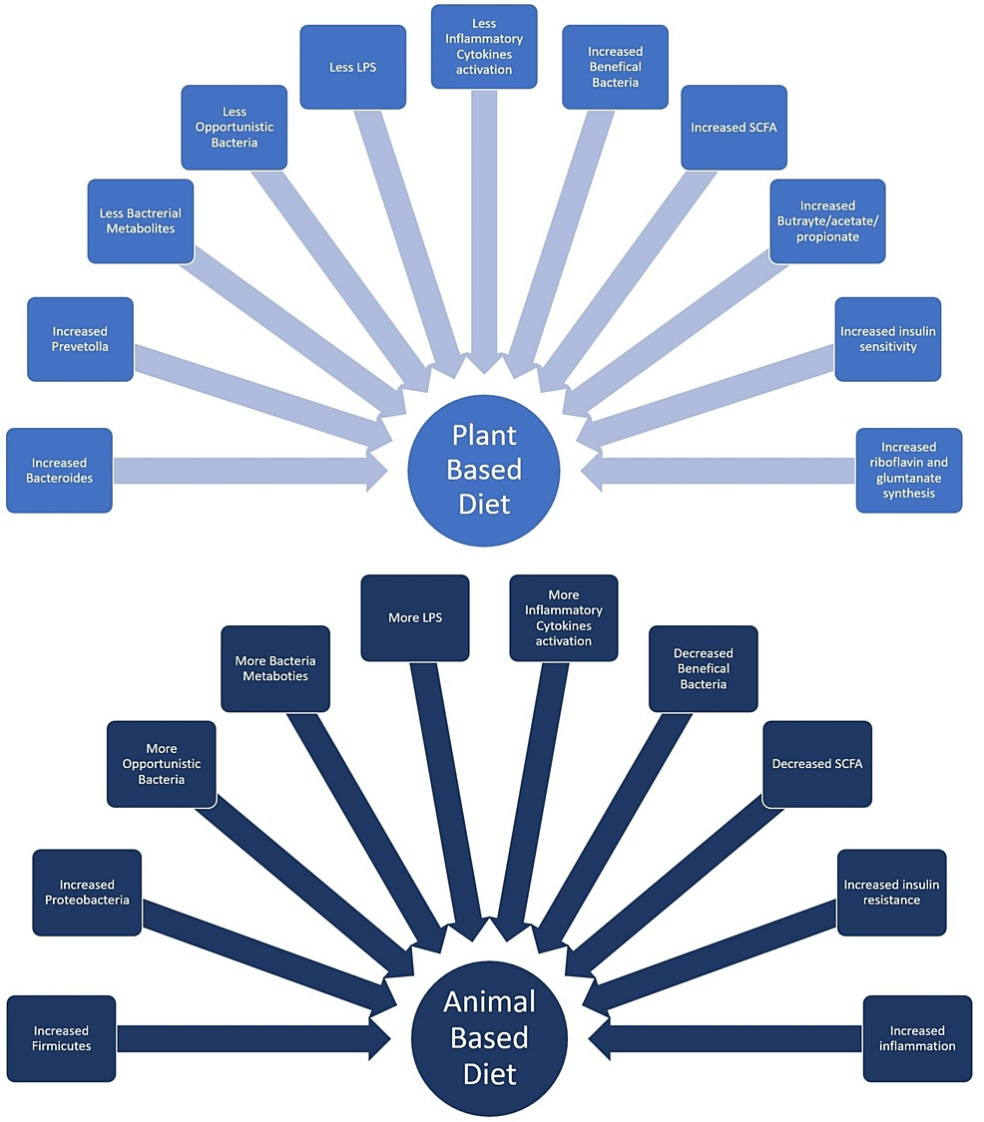
Plant-based diets (which are fiber-rich) vs. Animal-based diets (which have more fat and protein-rich components) have inverse relations in terms of inflammatory cytokine activation, LPS activation, SCFA activation, and beneficial and opportunistic bacteria composition, which is shown in detail. SCFA: Short-chain fatty acids, LPS: Lipopolysaccharides This figure is the author’s own creation.

The increased regulation of cellular mechanisms in inflammation, lipid absorption, and de novo lipogenesis may cause bile acids and cholesterol to function as emulsifiers [23]. Increased levels of lipopolysaccharides seen particularly in high-fat diet consumers have been connected to the activation of the TLR4 (Toll-like Receptor 4) receptor on macrophages and the production of more inflammatory markers, which has led to disruption of pancreatic beta-cell function and affected insulin sensitivity [23]. The flowchart in Figure 3 is a conceptual model developed to demonstrate the potential aspects associated with a plant-based diet vs. an animal-based diet and how they differ in their effects on the bacterial composition, activity, and cellular mechanisms in the intestinal microbiome [15,20].

According to a study by Candela et al., type 2 diabetes patients had more SCFA producers like *Faecalibacterium, Roseburia, Lachnospira, Bacteroides, *and* Akkermansia* in their gut microbiomes than healthy individuals who followed two different diet patterns (High-Fiber Diet vs. Control Diet) [15,27]. Results favored the high-fiber diet, not the control diet, which was successful in enhancing potential pro-inflammatory groups like *Collinsella* and *Streptococcus* in the gut ecosystem and demonstrated the potential to reverse pro-inflammatory dysbiosis in patients with type 2 diabetes, which may account for the higher efficacy in enhancing metabolic control. It has been demonstrated that *Bifidobacterium lactis*, seen in the high-fiber diet, enhances the translocation of glucose transport-4 and fosters glucose absorption [17].

Although observed findings regarding exercise have been noted in body composition and liver fat, the exact mechanism that modulates gut microbiota is unknown. It has been observed that SCFAs are changing, cholesterol metabolism is altered, and substrates for bacterial growth and gastrointestinal tract transit time are modified in the intestine [16].

*Prebiotics/Probiotics Supplementation* 

In clinical trials, a reduced number of *Bifidobacteria* was found to increase endotoxemia in high-fat diet-fed mice. With the supplementation of prebiotics, this was found to be reversed. In the plant-derived foods group, an increased synthesis of amylase, glutamate, and riboflavin was observed [16]. On the other hand, intake of animal-based foods leads to the adaptation of gut microbial function towards improved catabolic processes like the degradation of glycans and amino acids [20]. Gut bacteria called *Firmicutes* convert the enzyme flavin-containing monooxygenase 3 (FMO3), which produces trimethylamine N-oxide (TMAO). TMAO is also connected with type II diabetes, obesity, and inflammation [20].

Due to their ability to lower oxidative stress, probiotics have been identified as promising therapies for managing diabetes. In animal models and clinical trials, using probiotics to treat diabetes lowered fasting blood glucose and serum insulin levels. Probiotics, including *Bacteroides* and *Lactobacillus*, are being considered potential agents for the treatment of diabetes because of their role in increasing insulin sensitivity, lowering total cholesterol, and reducing body weight [18,19]. In animal investigations, *Bacteroides uniformis* and *Bacteroides acidifaciens* were also found to reduce insulin resistance and prevent obesity in diabetic mice. 

Through the insulin receptor substrate/phosphoinositide 3-kinase/protein kinase B signaling pathways, *Bifidobacterium*, also known as *Lactobacillus Bifidus*, is another potential prebiotic that is frequently used and observed in prediabetes and T2D and helps reduce blood glucose concentration, improve lipid profile, reduce insulin resistance, and increase antioxidant levels [19,20]. 

A typical probiotic strain of *Lactobacillus*, like *Lactobacillus casei*, prevents the release of endotoxins and activates the G-protein-coupled receptor 43 pathway. To maintain a healthy digestive system, *Lactobacillus,* and *Bifidobacterium* as supplementary probiotics have been utilized in combination, demonstrating the effectiveness of probiotic action at the microbial level and as a potential therapeutic approach [18,19]. *Actinoplanes* and *Lactobacillus* were found to efficiently block alpha-glucosidase activity, leading to lower glucose levels [29-31].

A prebiotic that has shown potential is the high-amylose maize starch used in a population of type 1 diabetic patients who were obese. It contained indigestible dietary fiber that helped increase SCFA production. Similarly, inulin, which is another probiotic used in type 1 diabetic patients, was found to promote more acetate production, which leads to SCFA production [21]. In metabolic syndrome (which is a group of metabolic abnormalities), with the development of prediabetes and type 2 diabetes, there was a decrease in inflammatory markers with the use of probiotics, but it was not as effective as when compared to antihyperglycemic drug therapy or healthy lifestyle changes [28,30,32]. There was evidence of a mild reduction in fasting glucose levels with probiotic supplementation in meta-analysis trials using randomized control trials. Another study showed that obese individuals using probiotic supplements had some weight loss, but it was not significant enough to be clinically relevant [22,33,31]. Another noteworthy fact is that many prebiotic/probiotic supplementation trials were not long enough to prove an association or reduction in metabolic factors [30,31]. Although there is evidence pointing to physiological gut microbial changes with prebiotic/probiotic supplementation, trials done to date have not shown a significant enough reduction in weight, insulin response as expected in clinical trials, or reduction in blood glucose levels [21]. Although a lot of positive correlation has been associated with prebiotics/probiotics in the mechanics of gut microbiome diversity and a healthy balance of phyla, the evidence thus far has not favored them as a substitute for antihyperglycemic therapy. There is insufficient evidence to conclude that dietary supplements reduce glycemic control. 

Changes in gut microbiome in metformin-treated groups and post-bariatric surgery patients

*Metformin* 

As a first-line agent to treat prediabetes and diabetes, lowering blood glucose levels while also acting on the liver, metformin given orally reaches the small intestine in smaller quantities [21]. It has been found to cause changes in gut microbiota by decreasing gluconeogenesis in part through modulation of mitochondrial complex I activity by the AMP-activated protein kinase pathway, a mechanism not fully understood [12,33]. Metformin works effectively by improving peripheral glucose sensitivity and reducing hepatic gluconeogenesis. Metformin has been associated with changes in SCFA, bile acids in the gut microbiota, and C-peptide levels, according to a study done by Lee et al. in 2021 [21]. Increasing SCFA can increase the secretion of GLP-1 levels, enhancing glucose-dependent insulin secretion [16,20]. Another study by Sun et al. and Ridlon et al. 2014 showed that metformin use in newly diagnosed type 2 diabetes patients reduced *Bacteroides fragilis* phylum and increased levels of bile acids, glycoursodeoxycholic acid [21].

In animal studies, metformin was found to act through multiple mechanisms in the gut microbiome, altering dysbiosis through unknown mechanisms [23,24]. Metformin seems to play a role in the alteration of *Lactobacillus* in both animal and human studies. In type 2 diabetic patients, clinical trials showed that Metformin usage changes the composition of the glucose hormone, other glucose-related parameters, and bile acid levels found in feces [24]. An important finding noted was the change in *Firmicutes* levels, which were found to be increased, and *Bacteroidetes* levels, which were found to be decreased after metformin treatment [21,22]. As already observed in type 2 diabetes, *Firmicutes/Bacteroidetes* proportions were found to be decreased, and this change in ratio was found to be increased with the treatment of metformin [24]. 

Metformin has also been found to impact glucose homeostasis by interfering with bile acid circulation, which functions by interacting with the intracellular nuclear receptor FXR (Farnesoid X receptor) signaling. Metformin works by interfering with bile acid resorption, resulting in increased gut exposure to bile acids and activating the FXR signaling mechanism. There have been significant associations between FXR signaling and enhanced insulin sensitivity and possibly with GLP-1 signaling, although the exact mechanism remains unknown [24]. Another important observation is the disruption of the mucus layer in the gut microbiota, which leads to bacteria interfering with gut permeability. *A. muciniphila* is a unique bacterium that degrades mucin while also stimulating more goblet cells that produce mucin [21]. In particular, the MUC2 and MUC5 genes were found to play an important role in mucin levels. Animal studies have revealed that the metformin-treated group showed more *A. muciniphila* by activating MUC2 genes, leading to more tight-junction proteins, zonulin-1 and occludin, involved in intestinal permeability [24]. The bile acid glycoursodeoxycholic acid was specifically increased after metformin administration. Additionally, these modifications revealed suppression of the intestinal farnesoid X receptor signaling mechanism in the metformin-treated group [32]. 

Although the precise mechanism of action of metformin is still unknown, its effects on the gut microbiome have been strongly demonstrated to be caused by an increase in short-chain fatty acids, a strengthening of intestinal permeability against lipopolysaccharides, modulation of the immune response, and interaction with bile acids [21,24]. Overall, metformin's positive change in glucose-lowering has been convincing, and the additional added benefit of increasing the therapeutic effects by supplementing with a plant-based diet, high-fiber diets, and the use of probiotics and prebiotics may be advantageous [24]. Metformin's action in the gastrointestinal tract may be an important factor in the development of gastrointestinal intolerance, which is a commonly reported adverse effect seen with drug usage. Metformin has been shown to increase glucose uptake, block mitochondrial oxidative phosphorylation, speed up glycolysis, and also increase lactate generation in enterocytes owing to its very effective role in targeting glycemia at the microbial level [12,33].

Bariatric Surgery

Bariatric surgery has the primary goal of long-lasting weight loss with restrictive food consumption. Various procedures, including vertical sleeve gastrectomy, Roux-en-Y bypass, and biliopancreatic diversion/duodenal switch, are available and being performed [25]. Reducing blood pressure, triglycerides, fasting glucose, increasing high-density lipoprotein, reducing waist circumference, reversing diabetes, and controlling glycemia have all been linked to bariatric surgery. Bile acids (BAs), hormonal shifts, changing dietary consumption, post-bariatric surgery, and variations in inflammatory metabolic factors were observed, all contributing to the transitions observed in the gut microbiota [25,29]. People with diabetes and obese individuals often have similar microbiota compositions, with lower microbial gene richness, lower microbial diversity, and a reduced Firmicutes-Bacteroidetes ratio [29,30]. In addition to the reduction of cardiometabolic risk with bariatric surgery, it has been found to impact gut microbiome diversity remarkably in post-bariatric surgery patients. A reduction of systemic and hepatic inflammation after bariatric surgery significantly impacted body weight and metabolism, and the effect on insulin signaling has been effectively modulated [25].

It was observed that bacteria that produced butyrate were increased in obese individuals and that there were decreased glycine levels in obese individuals, which was associated with an increased risk of developing type 2 diabetes. Similar to diabetic individuals, increased intestinal absorption of SCFAs via gene regulation was noted in obese individuals and was found to promote increased fat storage compared to non-obese individuals [25]. In obesity, there has been noted to be an increased permeability for bacterial toxins to cross the barrier, which has been associated with intestinal dysbiosis, low-grade inflammation, and insulin resistance. Thus, studies show that intestinal dysbiosis, which has only been found to be more active in those with a higher body mass index, is associated with the worsening of the disease progression associated with metabolic changes [25]. 

When comparing healthy and obese individuals, one can note a difference in the number of metabolites derived from gut microorganisms, such as higher production of aromatic amino acids (AAA) and branched-chain amino acids (BCAA). In the microbiota of obese individuals, the serum concentrations of phenylalanine, tyrosine, leucine, isoleucine, and valine were notably higher [25,32]. After bariatric surgery, there was an increase in lipopolysaccharides, leading to intestinal permeability. In obese individuals, there was more production of aromatic amino acids and branched-chain amino acids such as phenylalanine, tyrosine, leucine, isoleucine, and valine. Post-bariatric surgery, those who lost at least 10 kg were noted to have less branched-chain amino acid production in the gut, which could be another potential mechanism contributing to optimal glucose control [25]. In patients who underwent Roux-en-Y gastric bypass, an increase in oral bacteria such as *Fusobacteria*, *Veillonella*, and *Granulicatella* was observed, leading to pH changes directly affecting the gut microbiota diversity [25,29]. 

The hyperplasia of intestinal cells supports the hypothesis that post-gastric bypass surgery, a reduction in insulin resistance by increasing gluconeogenesis and improving the GLUT1 transporter levels was seen, which has been responsible for enhanced glucose uptake in tissues [25]. Following bariatric surgery, it was noted that there were changes in the bile acid signaling mechanisms in the physiological state that act through receptors FXR and TGR5 in the gut, impacting incretin hormones and glucose homeostasis [30,32]. Bile acid levels were discovered to be associated with enhanced and beneficial mechanisms in several key aspects, including satiety, cholesterol metabolism, incretin hormones, glucose homoeostasis, energy metabolism, gut microbiota, and endoplasmic reticulum stress in post-bariatric patients [29,32]. 

Fecal microbiota transplantation

Treatments that aim to turn a "dysbiotic" microbiota into a symbiotic one or increase beneficial bacteria in the gut microbiome have shown encouraging results. FMTs, when performed for a limited duration, showed moderate effects on metabolic disorders [26]. A very innovative way of targeting to change the composition of the intestinal microbiota was a process carried out in which feces was taken from healthy individual donors and transplanted into a recipient either via the duodenum or the colon infusion [26,27]. A study was done where the transfer of intestinal microbiota from lean donors was found to increase insulin sensitivity in which nine obese male subjects with metabolic syndrome were recruited for the study and received donor fecal microbial transplants, resulting in an improvement of insulin sensitivity and an increase in butyrate-producing microbiota after six weeks [28]. The same fecal microbiota transplant was done for lean subjects and showed increased bacterial diversity and the increased presence of butyrate-producing bacteria [26].

Another study done in 22 individuals with metabolic syndrome who underwent bariatric surgery (Roux-en-Y gastric bypass) and those with metabolic syndrome who were served after two weeks after fecal microbiota transplant revealed insulin sensitivity decreased more in individuals with metabolic syndrome recipients compared to the post-bariatric gastric bypass individuals, which showed reduced inflammation [27]. In the metabolic syndrome subjects in the study, there were changes noted in fecal bile acids, with increased lithocholic, deoxycholic, and (iso)lithocholic acid levels seen after FMT when compared to those who underwent post-bariatric surgery [27]. 

By altering glucagon-like peptide-1 (GLP-1), bile acid pathways, and short-chain fatty acid (SCFA) pathways, FMT has been shown to increase insulin sensitivity and is an innovative approach for resolving diseases in the gut microbiota by targeting to change the gut diversity, particularly in diabetes [22]. A few systematic analyses done on fecal microbial transplantation vs. placebo showed a small reduction in HBA1c levels, improved insulin sensitivity, and small changes in LDL and HDL cholesterol levels. As we lack enough evidence and need more participants to study these changes in clinical trials, we need further information and research into this form of therapy with a larger population undergoing the trial to know its full potential in therapeutics. It is premature to make assumptions about the management of diabetes with FMT without large and consistent evidence pointing to promising therapeutic oversight. However, the very possible observations from individuals tolerating the oral formulations and fecal microbiota transplant therapy done so far and the changes observed in the intestinal microbiome seem promising and revolutionary [21].

Limitations

These studies used in this review have several limitations because the studies included in this paper had relatively smaller study groups (the prebiotic supplementation trial and the FMT clinical trial), so the findings cannot be generalized ubiquitously to the entire population. The studies used in this review excluded newborns, children, gestational diabetes patients, and ICU patients. Even though the studies in this paper reflect that the usage of prebiotics, probiotics, and fecal microbial transplantation in diabetes management is plausible, data evaluating clear and consistent evidence regarding the positive impact of prebiotics, probiotics, and fecal microbial transplantation in diabetes is still lacking, and the quality of the evidence is still low. We only included studies published in the past 10 years. Also, studies whose free full texts were not accessible were not reviewed. The studies that were not included could have provided additional relevant information, which would have enhanced the quality of the review. Considering the favorable findings of the included studies, this review emphasizes the need for further, carefully conducted interventional studies to guide the practical use of dietary therapies that target the gut microbiota.

## Conclusions

The gut microbiota significantly had a strong rooted relationship in diabetic individuals, which showed consistent patterns of alterations in SCFA, modified bile acid metabolism, altered lipopolysaccharides, changes in bacterial compositions, and energy producers. The disruption of the normal colonic flora is the underlying problem that starts the cascade in the gut microbiome leading to dysbiosis. The efficacy of plant-based vs. animal-based diets showed differences in bacterial compositions and disproportionate ratios of the existence of bacterial phyla, which showed improved beneficial bacterial design and reduced the inflammatory cytokine activation, leading to improved insulin sensitivity. In small pilot studies, the use of prebiotics and probiotic supplementation showed reductions in inflammatory markers, which was not a substitute for drug therapy but did find mild improvements in insulin resistance, but not significant enough to warrant treatment in diabetic individuals. With the use of drugs like metformin and post-bariatric surgery, had microbial diversity has been noted and can help restore intestinal dysbiosis observed in diabetic individuals. Fecal microbial transplantation showed favorable gut mechanics, although extensive research in the field of gut microbiota is ongoing, with a real possibility for future prospects in therapeutics and the emergence of new forms of therapy. By further understanding the core pathophysiology and changes in the gut microbiome in metabolic diseases like diabetes and observing changes in disease patterns and inflammation, a revolutionary take on the management of metabolic diversity can be sought, which may pave the way for innovative therapeutic options in the near future. 
